# Bioaccessibility Study of Aflatoxin B_1_ and Ochratoxin A in Bread Enriched with Fermented Milk Whey and/or Pumpkin

**DOI:** 10.3390/toxins14010006

**Published:** 2021-12-22

**Authors:** Laura Escrivá, Fojan Agahi, Pilar Vila-Donat, Jordi Mañes, Giuseppe Meca, Lara Manyes

**Affiliations:** Laboratory of Food Chemistry and Toxicology, Faculty of Pharmacy, University of Valencia, 46100 Burjassot, València, Spain; Laura.Escriva@uv.es (L.E.); fojan@alumni.uv.es (F.A.); jordi.manes@uv.es (J.M.); giuseppe.meca@uv.es (G.M.); lara.manyes@uv.es (L.M.)

**Keywords:** bioaccessibility, aflatoxin B1, ochratoxin A, bread, pumpkin, whey, lactic acid bacteria

## Abstract

The presence of mycotoxins in cereals and cereal products remains a significant issue. The use of natural ingredients such as pumpkin and whey, which contain bioactive compounds, could be a strategy to reduce the use of conventional chemical preservatives. The aim of the present work was to study the bioaccessibility of aflatoxin B_1_ (AFB1) and ochratoxin (OTA) in bread, as well as to evaluate the effect of milk whey (with and without lactic acid bacteria fermentation) and pumpkin on reducing mycotoxins bioaccessibility. Different bread typologies were prepared and subjected to an in vitro digestion model. Gastric and intestinal extracts were analyzed by HPLC–MS/qTOF and mycotoxins bioaccessibility was calculated. All the tested ingredients but one significantly reduced mycotoxin intestinal bioaccessibility. Pumpkin powder demonstrated to be the most effective ingredient showing significant reductions of AFB1 and OTA bioaccessibility up to 74% and 34%, respectively. Whey, fermented whey, and the combination of pumpkin-fermented whey showed intestinal bioaccessibility reductions between 57–68% for AFB1, and between 11–20% for OTA. These results pointed to pumpkin and milk whey as potential bioactive ingredients that may have promising applications in the bakery industry.

## 1. Introduction

The most dangerous group of mycotoxins found in food are aflatoxins (AFs), produced by *Aspergillus* species. Among them, aflatoxin B_1_ (AFB1) is considered to be the most toxic mutagenic, teratogenic, and carcinogenic mycotoxin, which is classified as a group 1A (carcinogenic to humans) by the International Agency for Research on Cancer (IARC), and the most toxic compound by the European Commission [1,2]. AFs are commonly found in foodstuffs such as cereals and cereal-by products, corn, nuts, peanuts, coconut, dried fruits, and beer. On the other hand, ochratoxin A (OTA), produced by *Aspergillus* and *Penicillium* species, is also found in a wide variety of foodstuffs, mainly cereals and cereal-based products. The nephrotoxicity, immunotoxicity, mutagenicity, and neurotoxicity effects of OTA in humans has been proven by numerous studies [3]. Moreover, OTA is classified as group 2B, “possibly carcinogenic to humans” [1]. Maximum levels (MLs) of AFB1 and OTA have been established in different food products with values up to 4 and 5 μg/kg, respectively, for cereals and cereal-based products [2].

Despite the regulation of the MLs for both mycotoxins by the EU, several studies have demonstrated their presence in different cereals such as wheat and maize-derived products (bread, pasta, semolina, bulgur, cookie, infant foods etc.) reaching in worst case scenarios levels up to 150 µg/kg, exceeding the legal limits [4,5,6,7]. Bread and bakery products are the main foodstuff consumed around the world and are particularly important as a source of carbohydrates, proteins, and vitamins B and E [8]. However, it has been revealed that the baking process does not contribute to the reduction of mycotoxins levels. In fact, the effect of fermentation in bread only reduces OTA and AFB1 by 7% and 6% [9]. The high stability of OTA was confirmed as no significant change in its content was observed after fermentation and bread making process [10,11].

Different strategies have been developed to prevent or reduce mycotoxigenic fungal growth on food and feed by adding non-nutritional adsorbing agents [12,13,14,15]. 

Recently, there has been increasing interest in whether the absorption of mycotoxins in food could be reduced by microorganisms in the gastrointestinal tract. In light of this fact, one of the most used strategies in the reduction of mycotoxins bioaccessibility during the gastrointestinal digestion is the use of biocontrol agents. Certain lactic acid bacteria (LAB) strains were found to be able to remove different mycotoxins from foodstuffs by binding to their cell wall or by degradation with their enzymes in vitro [16,17,18,19]. In addition, the use of natural ingredients rich in bioactive compounds with antifungal properties (such as mustard flour or milk whey) are being studied nowadays as bio-preservatives in bread as response to the increasing consumers demand against the use of chemical additives [20,21,22,23].

Pumpkin, which belongs to the *Cucurbitaceae* family, is rich in carotenoids (i.e., lycopene, α- and β-carotene, lutein, and zeaxanthin) that play an important role in protecting cells from oxidation and cellular damage, preventing the incidence of human diseases such as mutagenic processes, cardiovascular diseases, osteoporosis, and diabetes [24]. Recent studies have shown that carotenoids-rich food such as pumpkin could counteract the toxic effects produced by mycotoxins [25,26]. Moreover, carotenoids revealed the ability of reducing mycotoxins such as AFB1 in rat tissues [27]. 

Milk whey is a cheese by-product in the dairy industry that has a high nutritional value and represents an important source of bioactive compounds, such as antifungal peptides [28]. The application of hydrolyzed goat milk whey as a bread ingredient improved the shelf-life and reduced mycotoxigenic fungal growth *(P. verrucosum),* as well as OTA production in pita bread [23]. Moreover, whey is also an optimum substrate for LAB fermentation and an excellent natural bio-preservative candidate in food production [29].

The aim of the present work was to evaluate the bioaccessibility of OTA and AFB1 when released from contaminated bread after a simulated in vitro human gastrointestinal digestion, as well as to study the effect on mycotoxins bioaccessibility of milk whey (with and without LAB fermentation), pumpkin, and the combination of both natural ingredients (pumpkin and whey) added to bread.

## 2. Results and Discussion

### 2.1. Bread Contamination and Analysis

Contaminated flour was analyzed in triplicated as previously explained. Barley flour showed OTA concentrations of 511 ± 84 mg/kg, while maize flour presented AFB1 levels of 6.2 ± 0.3 mg/kg. Based on those concentrations, contaminated breads with the single mycotoxin were prepared for each bread type; control (C), whey (W), fermented whey, (FW), pumpkin (P), fermented whey-pumpkin (FW-P) by adding 1.2 g of OTA contaminated flour or 10 g of AFB1 contaminated flour, aiming to obtain bread concentrations around 1000 µg/kg of OTA and 100 µg/kg of AFB1. Differences in both mycotoxins’ concentration are attributed to the natural contamination of cereals by-products that is usually much higher in the case of OTA than AFB1 [30,31]. 

Mycotoxin concentrations in the final bread were then analyzed by HPLC–MS/qTOF showing some differences among the bread typologies. As it is shown in Table 1, AFB1 bread concentrations ranged between 78–164 µg/kg, while OTA concentrations were between 1184–1540 µg/kg.

Several factors may affect the final bread composition (and in consequence, mycotoxin concentration) including nutrient concentration and changes in weight mainly due to water loss from the baking process. Depending on the ingredients of the initial dough, the baking process may be different, obtaining breads with different physicochemical properties. For example, whey addition as bread ingredient influenced rheological properties of wheat flour doughs reducing water absorption and increasing arrival time and dough development time [32]. On the other hand, fresh pumpkin that contains 80–96% moisture content, 4.6–6.5% sugars, 0.6–1.8% protein, 0.0–0.2% lipids, and 0.5–1.3% fiber, as well as other minor bioactive substances such as carotenoids, lose high amount of water in the lyophilization process, therefore nutrients are concentrated and bread enriched with dried pumpkin additive is expected to be richer in fiber, carotenoids, and other phytochemicals than the fresh vegetable. Moreover, pumpkin powder introduced as nutritional supplement was found to produce very large and unexpected increases in the loaf volume, as well as increase the organoleptic acceptability of wheat with comparatively poor bread making properties [33]. 

### 2.2. AFB1 Bioaccessibility

**Figure 1:**
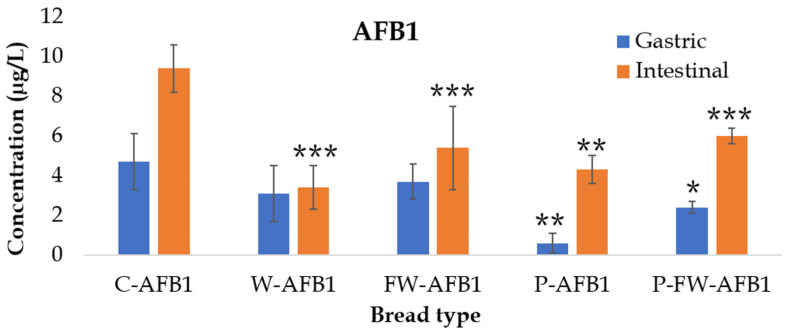
Gastric and intestinal concentration (µg/L) of aflatoxin B_1_ (AFB1) during the in vitro simulated digestion (n = 5). Significant differences from the control indicated as *p* < 0.05 (*), *p* < 0.01 (**), *p* < 0.001 (***). Control bread + AFB1 (C-AFB1); bread + whey + AFB1 (W-AFB1); bread + fermented whey + AFB1 (FW-AFB1); bread + pumpkin + AFB1 (P-AFB1); bread + fermented whey + pumpkin +AFB1 (FW-P-AFB1).

AFB1 release from bread during the simulated gastrointestinal digestion, as well as its bioaccessibility, was evaluated in control and enriched breads by the analysis of gastric and intestinal extracts. As it is shown in Figure 1, AFB1 concentration in gastric and intestinal extract from control bread (C-AFB1) was 4.7 ± 1.4 µg/L and 9.4 ± 1.2 µg/L, respectively, indicating a progressive mycotoxin release. However, gastric AFB1 concentration in enriched breads were between 0.6 ± 0.5 µg/L (P-AFB1) and 3.7 ± 0.9 µg/L (FW-AFB1), while in intestinal extracts it ranged between 3.4 ± 1.1 (W-AFB1), and 6.0 ± 0.4 µg/L (P-FW-AFB1). Enriched breads showed, in all cases, AFB1 concentrations lower than control bread for both gastric and intestinal phases. Significant differences from the control (*p* < 0.05) were observed for P-AFB1 and P-FW-AFB1 in the gastric extract, as well as in all enriched breads in the case of the intestinal concentrations.

The lower values obtained from AFB1 after gastric digestion may be due to the binding with long-chain carbohydrates, abundant in bread, which are hydrolyzed in duodenal digestion. In both gastric and intestinal extracts, the highest concentration was reached in the non-enriched bread (C-AFB1) giving a first indication that the natural added ingredients may have an effect on reducing the accessible fraction (bioaccessibility) of mycotoxins, hence reducing the amount of mycotoxin that could exert its effects at the gastrointestinal level.

The same trend was confirmed by the bioaccessibility results, where the highest values were achieved for non-enriched bread (C-AFB1) with gastric and intestinal bioaccessibility of 61 ± 19% and 114 ± 9%, respectively. Bioaccessibility values higher than 100% have been previously reported for mycotoxins in processed cereal-based food samples, and it could be attributed to possible interactions established between food matrix, mycotoxins, and digestive fluids [34,35]. Table 2 shows AFB1 gastric and intestinal bioaccessibility (%) from bread with and without enrichment ingredients. As it is shown, all enriched breads (W, FW, P, and FW-P) managed to reduce AFB1 bioaccessibility in some extent, and breads enriched with pumpkin showed the lowest bioaccessibility (4 ± 2 % and 29 ± 5 %) in gastric (*p* ≤ 0.05) and intestinal (*p* ≤ 0.0001) extracts, respectively, followed by bread enriched with the combination of fermented whey and pumpkin (15 ± 2% and 37 ± 3%, respectively). Since several studies have suggested the use of LAB fermentation, in order to bind AFB1 and reduce its gastrointestinal bioaccessibility [17,18,23,36,37] milk whey additive with and without previous LAB fermentation was examined in order to determine whether there could be any reduction on AFB1 bioaccessibility due to the bioactive compounds generated during fermentation. As shown in Table 2, enrichment treatments with W and FW showed lower AFB1 intestinal bioaccessibility (41–52%) compared to control bread (114%) (Table 2).

Data about AFs bioaccessibility is valuable to mitigate chronic hazards, mainly regarding food and feed that are frequently consumed; also, mycotoxins are thermal-resistant compounds, making it difficult to mitigate their presence in food and feed by conventional processes that apply moderated conditions such as cooking, extrusion, and baking. Studies concerning the bioaccessibility of AFB1 are scarce. Similarly to the obtained results, AFB1 bioaccessibility was between 83–108% for peanut slurry [35], between 85 and 98%, for various spiked food matrices (peanut, pistachio, hazelnut, dried figs, paprika, wheat, and maize) [38]; and 85% in fish feed [39]. Conversely, lower values were obtained for AFB1 bioaccessibility in spiked loaf bread during the stomach and the duodenal digestion reaching 54% and 26%, respectively [17]. It should be noted that food matrix, contamination level, compound, and type of contamination (spiked versus naturally contaminated) and even the gastrointestinal digestion model used can interfere with mycotoxin bioaccessibility performances as well [37]. 

There are no available studies assessing pumpkin (or carotenoids-rich food) and whey on AFB1 bioaccessibility reduction. However, the protective effect of pumpkin extract against AFB1 toxicity and *Aspergillus flavus* induced lung damage was reported in rats, effect that may be related to the antioxidant constituents of pumpkin extract [40]. 

Along the same line, other authors demonstrated that the antioxidant compounds of grape seed meal counteracted AFB1 toxicity in piglet mesenteric lymph nodes, showing a reduction of AFB1-induced oxidative stress by increasing the activity of glutathione peroxidase (GPx) and superoxide dismutase (SOD) and decreasing lipid peroxidation [41]. Supplementing the diet of ducks with antioxidants such as curcumin increased antioxidant capacity, inhibiting lipid and protein oxidation in their meat [42]. Moreover, the protective effects of lycopene against the toxic effects of AFB1 in kidney and heart were evaluated in rats [27], and the cellular and molecular mechanisms by which the antioxidants β-carotene and lycopene inhibit AFB1-induced toxic changes were also investigated in several cell lines, including Hep-G2 (Hep G2) cells [43].

On the other hand, Ferrer et al. [44] investigated the influence of some food ingredients (including milk whey, β-lactoglobulin, and calcium caseinate) and probiotic microorganisms on the bioaccessibility of deoxynivalenol (DON), zearalenone (ZEN), beauvericin (BEA), and enniatins (ENs A, A1, B, B1) in wheat crispy breads concluding that the addition of prebiotics and bioactive microorganisms decreased the bioaccessibility of mycotoxins, with a concentration-dependent behavior [44].

### 2.3. OTA Bioaccessibility

**Figure 2:**
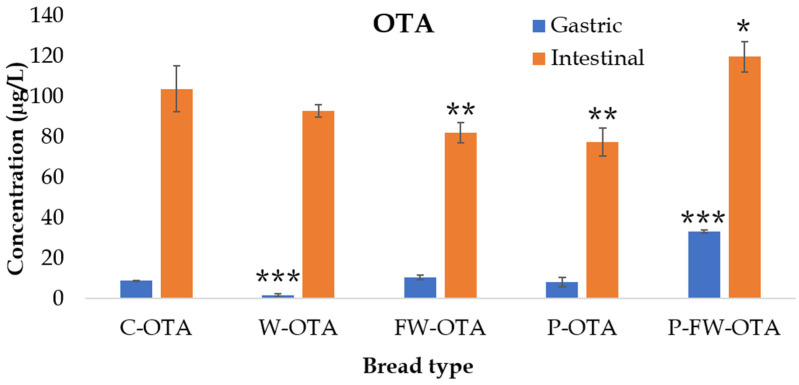
Gastric and intestinal concentration (µg/L) of ochratoxin A (OTA) during the in vitro simulated digestion (n = 5). Significant differences from the control indicated as *p* < 0.05 (*), *p* < 0.01 (**), *p* < 0.001 (***). Control bread + OTA (C-OTA); bread + whey + OTA (W-OTA); bread + fermented whey + OTA (FW-OTA); bread + pumpkin + OTA (P-OTA); bread + fermented whey + pumpkin + OTA (FW-P-OTA).

Regarding OTA gastrointestinal digestion, it was observed that concentrations in the gastric compartment were considerably lower than those detected in the intestinal stage (Figure 2), indicating that OTA release from bread mainly occurs at the intestinal digestion step with an important effect of medium pH. OTA consists of a dihydroisocoumarin moiety linked through its 7-carboxyl group by an amide linkage to L-phenylalanine. The pKa values are in the ranges of 4.2–4.4 and 7.0–7.3, respectively, for the carboxyl group of the phenylalanine moiety and the phenolic hydroxyl group of the isocoumarin part. This indicates that, in aqueous solutions near pH 7, both the monoanion (OTA−) and the dianion (OTA^2^−) are present, whereas the molecular toxin is prevalent in acid solutions (pH < 3). This would cause the mycotoxin to be released with pH medium changes from gastric to intestinal conditions. In fact, numerous studies in which OTA adsorption (by adsorbing agents) was investigated by in vitro gastrointestinal digestions showed that OTA adsorption was significantly affected by the pH at acid gastric conditions [13,45].

Gastric and intestinal OTA concentrations in control bread (C-OTA) were 9.1 ± 0.1 and 103.8 ± 11.3 g/L, respectively. Gastric OTA concentrations in enriched breads were significantly lower (*p* < 0.05) in the case of whey and pumpkin-fermented whey additives, while lower intestinal OTA concentrations were observed in fermented whey and pumpkin enriched breads (Figure 2). OTA gastric concentrations in enriched breads ranged between 1.8 ± 0.6 µg/L (W) and 33.3 ± 0.9 µg/L (P-FW), while intestinal digests showed concentrations from 77.5 ± 7.0 (P) to 119.7 ± 7.7 µg/L (P-FW). The lowest OTA intestinal concentration was found for pumpkin treatment, however, in the gastric extract the lowest OTA levels were found for whey, followed by pumpkin enrichments (Figure 2).

OTA bioaccesibility was examined with and without enriched ingredients, showing gastric and intestinal bioaccessibility in the control bread of 7.7 ± 0.1% and 88 ± 10%, respectively (Table 3). This difference may be explained since OTA can be released when the medium pH increases up to 7 (intestinal conditions), due to the physical-chemical properties of this molecule, as previously discussed. Previous works demonstrated high OTA bioaccessibility (100%) in naturally contaminated buckwheat [35] and lower bioaccessibility in naturally (22%) and artificially contaminated infant food (29–32%) [37], suggesting that bioaccessibility depends on several factors, such as food product, contamination level, compound, and type of contamination [34,38,46]. 

With regard to the enriched breads, gastric bioaccessibility values found in the present study were between 1.4 ± 0.5% (W-OTA) and 21.6% (P-FW-OTA), being statistically lower than the control in the case of whey- and pumpkin-enriched breads. Intestinal bioaccessibility of enriched breads was in all cases lower than in the control, ranging from 58 ± 5% (P-OTA) to 74 ± 2% (W-OTA), with significant difference in all enrichments but the combination pumpkin-fermented-whey (P-FW-OTA). Similarly, as for AFB1, the lowest OTA intestinal bioaccessibility was found for pumpkin, while the lowest OTA gastric bioaccessibility was found for whey, followed by pumpkin enrichments (Table 3).

There are no available studies assessing the effect of pumpkin and whey on reducing OTA bioaccessibility in vitro. However, the detoxification potential of whey powder against OTA harmful effects was investigated in broilers showing significant reduction of the hematobiochemical parameters raised by OTA treatment, as well as reduction in OTA residues detected in several organs including kidney, suggesting the potential application of whey ingredient in broiler feeds to reduce the negative effects of OTA in animals as efficiently as commercial mycotoxin binders [47]. Moreover, whey supplementation showed a vital role in maintaining the integrity of liver and kidney functions in fish exposed to OTA, ameliorating animals’ performance, histopathological alterations, and biochemical parameters, and demonstrating its protective role against OTA toxicity especially with low dose [48,49].

### 2.4. Effect of Bread Enrichment with Bioactive Ingredients on Mycotoxin Reduction

This is the first work in which these natural ingredients have been added to bread formulation, alone and in combination, to study AFB1 and OTA bioaccessibility and their efficacy in reducing mycotoxins bioaccessibility. In the present work, pumpkin extract demonstrated to be the most effective treatment applied in bread, with significant intestinal bioaccessibility reductions (*p* < 0.0001) of both mycotoxins. AFB1 bioaccessibility decreased from 114% in control bread, up to 29% in bread enriched with pumpkin (P) (Table 2). Moreover, pumpkin enrichment revealed the major effect on reducing OTA intestinal bioaccessibility with significant reductions (*p* < 0.0001) from 88% in control bread up to 58% in bread enriched with pumpkin (P) (Table 3). This means AFB1 and OTA reductions of 74% and 34%, respectively, when pumpkin is present in bread formulation (Table 4).

Whey additive also showed significant reductions (*p* < 0.0001) of AFB1 bioaccessibility, from 114% in control bread, up to 41% and 52% in bread enriched with whey (W) and fermented whey (FW), respectively (Table 2). However, in the case of OTA, bioaccessibility values decreased from 88% in control bread up to 74% (W) and 70% (FW) in enriched breads (Table 3). Therefore, AFB1 and OTA intestinal bioaccessibility reductions between 57–64% and 16–20%, respectively, were achieved compared to control breads without preservatives (Table 4). However, no high differences were observed when both ingredients were combined (FW-P), reaching AFB1 and OTA bioaccessibility reductions of 68% and 11% for each mycotoxin, respectively (Table 4).

## 3. Conclusions

In this study, mycotoxins (AFB1 and OTA) bioaccessibility in bread enriched with milk whey and pumpkin has been studied by an in vitro digestion model. The obtained results indicate that almost all enrichment treatments significantly reduced AFB1 and OTA bioaccessibility compared to the control bread without preservatives. Interesting reductions were observed for milk whey; however, pumpkin seems to be the most efficient ingredient on reducing mycotoxin bioaccessibility. Hence, the addition of pumpkin and whey in the bread making process could be a strategy to reduce the absorbable fraction of mycotoxins at the intestinal level. Therefore, they can be potential candidates as bioactive ingredients for bread formulation. 

One of the main challenges in the food technology field is to develop new bio-preservatives that would support detoxification in a broad spectrum of food matrices. These ingredients could be used along with other biocontrol agents to counteract AFB1 and OTA in bakery products.

In addition, these natural ingredients introduced in bread dough at levels (1%) that can be applied in the industrial bread-making process could produce increases in the loaf volume, as well as improve the organoleptic acceptability and breads’ shelf-life, potentially enriching their nutritional value as previously shown in the literature. Therefore, their potential application at the industry level is very feasible and should be further studied considering their organoleptic and nutritional qualities, but a larger sample size should be included.

The determination of mycotoxins bioaccessibility by in vitro methods offers an appealing alternative to human and animal studies avoiding the use of more complex cell culture techniques or the use of animals in expensive in vivo experiments, complying with the principle of the “3Rs”: reducing, reusing, and recycling resources. However, despite the wide applications of these static in vitro digestion models, limitations such as oversimplify the digestive physiology and failing to mimic the dynamic aspects of the digestive process should be considered when interpreting the data.

## 4. Material and Methods

### 4.1. Chemicals, Reagents and Biological Strains

AFB1 and OTA standard solutions (purity > 99%) were purchased from Sigma-Aldrich (St. Louis, MO, USA). Potassium chloride (KCl), potassium thiocyanate (KSCN), sodium dihydrogen phosphate (NaH_2_PO4), sodium sulfate (Na_2_SO_4_), sodium chloride (NaCl), sodium hydrogen carbonate (NaHCO3), urea (CO(NH_2_)_2_), α-amylase (930 U mg^−1^ A3403), hydrochloric acid (HCl), sodium hydroxide (NaOH), formic acid (HCOOH), pepsin A (674 U mg^−1^ P7000), pancreatin (762 U mg^−1^ P1750), bile salts (B8631), and phosphate buffer saline (PBS, pH 7.5) were purchased from Sigma-Aldrich (Madrid, Spain). Methanol and ethyl acetate were supplied by Fisher Scientific (Madrid, Spain). Deionized water was purchased from a Milli-Q water purification system (Millipore, Bedford, MA, USA). LAB used in this study (*L. plantarum CECT 220*) was obtained from the Spanish Type Culture Collection, CECT, Science Park of the University of Valencia (Paterna, València, Spain). The cultures were kept at −80 °C in glycerol 25% until their use.

### 4.2. Flour Contamination

Barley and maize flour were naturally contaminated by the fungal species-producers of AFB1 and OTA *Aspergillus steynii 20510* (obtained from CECT) and *A. flavus ITEM 8111* (obtained from the Agro-Food Microbial Culture Collection of The Institute of Sciences and Food Production (ISPA, Bari, Italy)), respectively. To that aim, 300–350 g of maize or barley were introduced in 1 L glass jars previously autoclaved. Then, cereals were contaminated with 15–20 mL of spores and mycelium suspension in peptone water with Tween 80 (0.1% both) of the corresponding fungal strain. Glass jars were then left at room temperature in darkness for one month. After that, cereals were autoclaved to remove the fungal contamination and samples were ground to flour until complete homogenization. Mycotoxins in contaminated flour were quantified by high performance liquid chromatography coupled to time-of-flight mass spectrometry (HPLC–MS/qTOF) after a solid-liquid extraction, as explained below in Section 4.5.

### 4.3. Bread Natural Ingredients

Milk whey strained from goat’s milk coagulated by commercial rennet (starter culture R-604) was obtained from the ALCLIPOR society, S.A.L. (Benassal, Spain). Milk whey ingredient was studied with and without LAB fermentation. For milk whey fermentation, 4 mL of LAB suspension at concentration of 10^8^ CFU/mL were added to 40 mL milk whey, previously pasteurized in accordance with standardized guidelines [50], and samples were incubated 72 h at 37 °C to allow LAB fermentation. Fermented and non-fermented whey were then lyophilized to obtain a homogeneous powder.

Pumpkin was obtained from a commercial supermarket in Valencia (Spain). Pumpkin powder was prepared by pealing and cutting the fresh vegetable (skin and seeds previously removed) followed by lyophilization and grinding to obtain a homogeneous powder. Both ingredients were analyzed to confirm the absence of mycotoxins, and stored at −20 °C until their use.

### 4.4. Bread Preparation and Baking

Fifteen breads were prepared combining the bioactive ingredients with and without the studied mycotoxins, OTA and AFB1. Control bread preparation was performed by the following recipe: 300 g of wheat flour, 165 mL of mineral water (37 °C), 20 g of yeast for bakery products (Levital, Spain), 10 g of sucrose, and 6.5 g of NaCl. After mixing all the ingredients, doughs were homogenized in a bakery machine (Silver Crest) for 5 min, shaped in molds (100 g), covered with a damp cloth, and left 1 h to ferment at room temperature. After that, breads were covered with silver foil and baked at 200 °C for 45 min in a Memmert ULE 500 muffle furnace (Madrid, Spain). Finally, breads were unmolded and cooled at room temperature (1 h). Enriched breads were then prepared by slight modifications on the control recipe obtaining a) control bread (C), b) 1% milk whey bread (W), c) 1% of fermented milk whey bread (FW), d) 1% of lyophilized pumpkin bread (P), and e) 1% of fermented milk whey + 1% of lyophilized pumpkin bread (FW-P). Then, contaminated breads with OTA and AFB1 were prepared for the five bread conditions (C, W, FW, P, FW-P) substituting a fraction of wheat flour by 1.2 g of barley flour contaminated with OTA and/or 10 g of maize flour contaminated with AFB1. Fifteen breads were obtained, three for each enrichment type, as shown in Table 5.

### 4.5. Bread Analysis

For bread analysis the method described by Saladino et al. [5] was followed. Briefly, 5 g of bread were accurately weighed (precision 0.1 mg), transferred to centrifuge tubes (50 mL), and 25 mL of methanol were added. Samples were crushed in Ultraturrax (T 18 digital ULTRA-TURRAX^®^, Staufen, Germany) for 5 min and centrifuged at 4500 rpm for 10 min (Centrifuge 5810R, Eppendorff, Germany). Supernatant was collected in new centrifuge tubes and evaporated until complete dryness using a rotavapor (BUCHI Rotavapor R-200, Postfach, Switzerland) and turbovap (TurboVap LV Evaporator, Zymark, Hopkinton, MA, USA). Samples were then reconstituted in 1 mL of methanol, filtered with a 0.22-μm filter (Phenomenex, Madrid, Spain) and injected for HPLC–MS/qTOF analysis. All the analyses were performed by triplicate (n = 3).

### 4.6. HPLC–MS/qTOF Conditions

Chromatographic analysis was performed on an Agilent 1200 (Agilent Technologies, Santa Clara, CA, USA), consisting of an auto sampler, vacuum degasser, and binary pump. Analyte separation was carried out using a Gemini C18 column (50 mm × 2 mm, 110 Å and particle size 3 μm; Phenomenex, (Phenomenex, Palo Alto, CA, USA)). The mobile phases consisted of water (solvent A) and acetonitrile (solvent B) both with 0.1% of formic acid with an elution flow rate of 0.3 mL/min and an elution gradient as follows: 0 min, 5% B; 30 min, 95% B; 35 min, 5% B. Total analysis run was achieved in 25 min and the injection volume was 5 µL. For mass spectrometry analyses, a MS/qTOF (6540 Agilent Ultra High-Definition Accurate Mass, Agilent Technologies, Santa Clara, CA, USA), coupled to an Agilent Dual Jet Stream electrospray ionization (Dual AJS ESI) interface operating in positive ion mode was used. Optimized mass spectrometry parameters included: fragment voltage 175 V; capillary voltage 3.5 kV; collision energy 10, 20 and 40 eV, nebulizer pressure 30 psi; drying gas flow (N_2_) 8 L/min, temperature 350 °C. Data analysis was performed by MassHunter Qualitative Analysis Software B.08.00. (Agilent Technologies, Santa Clara, CA, USA).

### 4.7. In Vitro Static Digestion Model

To reproduce the complete process of the human digestion, an in vitro digestion model was applied based on Manzini et al. (2015) [51], with some modifications. Briefly, 10 g of bread were placed in sterilized plastic bags (500 mL), mixed with 6 mL of artificial saliva and 80 mL of Milli-Q water (37 °C) (Millipore, Bedford, MA, USA). To replicate the oral phase, an IUL Stomacher (IUL S.A, Barcelona, Spain) was applied for 30 s simulating mastication process. Saliva was prepared the day before (and adjusted to pH = 6.8) by mixing 10 mL of KCl (89.6 g/L), 10 mL of KSCN (20 g/L), 10 mL of NaH_2_PO_4_ (88.8 g/L), 10 mL of Na_2_SO_4_ (57 g/L), 1.7 mL of NaCl (175.3 g/L), 20 mL NaHCO_3_ (84.7 g/L), 8 mL of urea (25 g/L), 290 mg of α-amylase, and 430 mL of distilled water. After simulation of the oral phase, the content was transferred to a topaz Erlenmeyer flask to continue with the gastric phase. The mixture was acidified to pH = 2 with 6N HCl solution. Then 0.5 g of pepsin solution (1 g in 25 mL of 0.1N HCl) and 14 mL of Milli-Q water (37 °C) were added to complete the final volume to 100 mL. Samples were incubated 2h at 37 °C under darkness and slight agitation (100 rpm) using an orbital shaker (Infors AG CH-4103, Bottmingen, Switzerland). After that, gastric aliquots (15 mL) were kept for analysis and pancreatic digestion was reproduced by adding 1.25 g bile salts/pancreatin mixture (0.1 g pancreatin and 0.625 g of bile salts dissolved in 25 mL of 0.1N NaHCO_3_), at pH = 6.5 (0.5N NaHCO_3_). Extracts were incubated as previously (2 h at 37 °C in darkness and slight agitation), pH was finally adjusted to 7.2 (0.5N NaOH), samples were centrifuged (4500 rpm for 10 min at 4°C), and supernatant was collected obtaining the intestinal digested extracts. All the digestions were performed with five replicates (n = 5).

### 4.8. Gastrointestinal Extracts Analysis and Bioaccessibility

Gastric aliquots and intestinal extracts were filtered (0.22 μm filter) and directly injected in the HPLC–MS/qTOF for mycotoxins determination. Standard calibration curves were prepared in methanol (1–1000 µg/L) from OTA and AFB1 standards (1000 mg/L). For quantitation purposes, matrix-matched calibration curves were prepared for all bread conditions (C, W, FW, P, FW-P) by spiking blank digested extracts with OTA and AFB1 at the same concentrations.

Mycotoxins bioaccessibility (%) was calculated as the percentage of mycotoxins from the initial digested bread that were detected in the digested extracts. The mycotoxin quantity (μg) in 10g of bread (A) was calculated from bread concentration (μg/kg) by conversion factors (×10/1000). The mycotoxin quantity (μg) in 100 mL of digest (B) was calculated from digest concentration (μg/L) by conversion factors (×100/1000). To calculate the bioaccessibility percentage, both quantities were related as B/A×100.

Combining all the calculations, the bioaccessibility could be directly calculated as indicated in Equation (1):

(1) $$\mathrm{Bioaccesibility}=\frac{\mathrm{digest}\;\mathrm{concentration}\;\left(\frac{\mu g}{L}\right)\times 1000}{\mathrm{bread}\;\mathrm{concentration}\;\left(\frac{\mu g}{\mathrm{kg}}\right)}$$

Statistical analysis was performed by a Student’s repeated measures *t*-test (n ≥ 3) to analyze all the results considered as significant, *p* values < 0.05.

## Figures and Tables

**Table 1:** Aflatoxin B1 (AFB1) and ochratoxin (OTA) concentration (µg/kg) in the prepared breads. Average results and standard deviation of triplicate samples (n = 3).

		Bread Concentration
		(µg/kg)
AFB1	C-AFB1	78 ± 3
	W-AFB1	92 ± 12
	FW-AFB1	85 ± 6
	P-AFB1	148 ± 5
	FW-P AFB1	164 ± 16
OTA	C-OTA	1184 ± 118
	W-OTA	1258 ± 220
	FW-OTA	1173 ± 78
	P-OTA	1336 ± 202
	FW-P-OTA	1540 ± 291

Control bread + AFB1 (C-AFB1); bread + whey + AFB1 (W-AFB1); bread + fermented whey + AFB1 (FW-AFB1); bread + pumpkin + AFB1 (P-AFB1); bread + fermented whey + pumpkin +AFB1 (FW-P-AFB1); control bread + OTA (C-OTA); bread + whey + OTA (W-OTA); bread + fermented whey + OTA (FW-OTA); bread + pumpkin + OTA (P-OTA); bread + fermented whey + pumpkin + OTA (FW-P-OTA).

**Table 2:** Gastric and intestinal bioaccessibility (%) of AFB1 in bread (C), and enriched breads with milk whey (W), fermented milk whey (FW), pumpkin (P), and fermented milk whey + pumpkin (P-FW). Significantly different from the control, *p* ≤ 0.05 (*), *p* ≤ 0.0001 (***). Mean ± standard deviation (n = 5).

Bread Type	Gastric Bioaccessibility (%)	Intestinal Bioaccessibility (%)
C-AFB1	61 ± 19	114 ± 9
W-AFB1	34 ± 15	41 ± 13 ***
FW-AFB1	43 ± 11	52 ± 5 ***
P-AFB1	4 ± 2 *	29 ± 5 ***
P-FW-AFB1	15 ± 2 *	37 ± 3 ***

Control bread + AFB1 (C-AFB1); bread + whey + AFB1 (W-AFB1); bread + fermented whey + AFB1 (FW-AFB1); bread + pumpkin + AFB1 (P-AFB1); bread + fermented whey + pumpkin +AFB1 (FW-P-AFB1).

**Table 3:** Gastric and intestinal bioaccessibility (%) of OTA in bread (C), and enriched breads with milk whey (W), fermented milk whey (FW), pumpkin (P), and fermented milk whey + pumpkin (P-FW). Significantly different from the control, *p* ≤ 0.05 (*), *p* ≤ 0.001 (**), *p* ≤ 0.0001 (***). Mean ± standard deviation (n = 5).

Bread Type	Gastric Bioaccessibility (%)	Intestinal Bioaccessibility (%)
C-OTA	7.7 ± 0.1	88 ± 10
W-OTA	1.4 ± 0.5 **	74 ± 2 *
FW-OTA	9.0 ± 1.1	70 ± 4 *
P-OTA	6.1 ± 1.8	58 ± 5 ***
P-FW-OTA	21.6 ± 0.6 ***	78 ± 5

Control bread + OTA (C-OTA); bread + whey + OTA (W-OTA); bread + fermented whey + OTA (FW-OTA); bread + pumpkin + OTA (P-OTA); bread + fermented whey + pumpkin + OTA (FW-P-OTA).

**Table 4:** Intestinal bioaccessibility reduction (%) of AFB1 and OTA in bread enriched with milk whey (W), fermented milk whey (FW), pumpkin (P), and fermented milk whey + pumpkin (P-FW) compared to the control bread.

Bread Ingredient	Intestinal Bioaccessibility Reduction (%)	
	AFB1	OTA
W bread	64 ± 11	16 ± 3
FW bread	57 ± 4	20 ± 5
P bread	74 ± 4	34 ± 6
P-FW bread	68 ± 2	11 ± 6

W bread (bread enriched with whey), FW bread (bread enriched with fermented whey), P bread (bread enriched with pumpkin), and P-FW bread (bread enriched with pumpkin+ fermented whey).

**Table 5:** Bread conditions prepared in the present study. Whey concentration (1%), fermented whey concentration (1%), pumpkin concentration (1%).

Bread Type
**Control Bread**
Bread (C)
Bread + AFB1 (C-AFB1)
Bread + OTA (C-OTA)
**Whey bread**
Bread + whey (W)
Bread + whey + AFB1 (W-AFB1)
Bread + whey + OTA (W-OTA)
**Fermented whey bread**
Bread + fermented whey (FW)
Bread + fermented whey + AFB1 (FW-AFB1)
Bread + fermented whey + OTA (FW-OTA)
**Pumpkin bread**
Bread + pumpkin (P)
Bread + pumpkin + AFB1 (P-AFB1)
Bread + pumpkin + OTA (P-OTA)
**Fermented whey-Pumpkin bread**
Bread + fermented whey + pumpkin (FW-P)
Bread + fermented whey + pumpkin +AFB1 (FW-P-AFB1)
Bread + fermented whey+ pumpkin + OTA (FW-P-OTA)

## Data Availability

Data are available upon request; please contact the contributing authors.
